# The Effect of Different Water Immersion Temperatures on Post-Exercise Parasympathetic Reactivation

**DOI:** 10.1371/journal.pone.0113730

**Published:** 2014-12-01

**Authors:** Vinícius de Oliveira Ottone, Flávio de Castro Magalhães, Fabrício de Paula, Núbia Carelli Pereira Avelar, Paula Fernandes Aguiar, Pâmela Fiche da Matta Sampaio, Tamiris Campos Duarte, Karine Beatriz Costa, Tatiane Líliam Araújo, Cândido Celso Coimbra, Fábio Yuzo Nakamura, Fabiano Trigueiro Amorim, Etel Rocha-Vieira

**Affiliations:** 1 Laboratório de Biologia do Exercício, Centro Integrado de Pesquisa em Saúde, Programa Multicêntrico de Pós-Graduação em Ciências Fisiológicas – Universidade Federal dos Vales do Jequitinhonha e Mucuri, Diamantina, Brazil; 2 Núcleo de Estudos em Reumatologia, Esportiva e Recursos Terapêuticos – Universidade Federal de Santa Catarina, Araranguá, Brazil; 3 Laboratório de Endocrinologia, Instituto de Ciências Biológicas, Universidade Federal de Minas Gerais, Belo Horizonte, Brazil; 4 Departamento de Educação Física, Universidade Estadual de Londrina, Londrina, Brazil; University of Buenos Aires, Faculty of Medicine. Cardiovascular Pathophysiology Institute, Argentina

## Abstract

**Purpose:**

We evaluated the effect of different water immersion (WI) temperatures on post-exercise cardiac parasympathetic reactivation.

**Methods:**

Eight young, physically active men participated in four experimental conditions composed of resting (REST), exercise session (resistance and endurance exercises), post-exercise recovery strategies, including 15 min of WI at 15°C (CWI), 28°C (TWI), 38°C (HWI) or control (CTRL, seated at room temperature), followed by passive resting. The following indices were assessed before and during WI, 30 min post-WI and 4 hours post-exercise: mean R-R (mR-R), the natural logarithm (ln) of the square root of the mean of the sum of the squares of differences between adjacent normal R–R (ln rMSSD) and the ln of instantaneous beat-to-beat variability (ln SD1).

**Results:**

The results showed that during WI mRR was reduced for CTRL, TWI and HWI versus REST, and ln rMSSD and ln SD1 were reduced for TWI and HWI versus REST. During post-WI, mRR, ln rMSSD and ln SD1 were reduced for HWI versus REST, and mRR values for CWI were higher versus CTRL. Four hours post exercise, mRR was reduced for HWI versus REST, although no difference was observed among conditions.

**Conclusions:**

We conclude that CWI accelerates, while HWI blunts post-exercise parasympathetic reactivation, but these recovery strategies are short-lasting and not evident 4 hours after the exercise session.

## Introduction

Physical exercise induces changes in different physiological systems that depend on exercise mode, duration and intensity [1]. To cope with the metabolic demand imposed by exercise, the cardiovascular system responds by increasing cardiac output, observed by increases in stroke volume and heart rate (HR). These changes are modulated by parasympathetic withdrawal and increased sympathetic stimulus to sinus node [2], [3].

In the post exercise period, the body must recover from the exercise stress. Recovery is a process of restoration of physical performance to a former or higher level. It involves the integrated response of many systems that return the body to its prior homeostasis or to a higher level of homeostasis [4]. The cardiovascular system plays a fundamental role during the recovery process, as it facilitates many physiological processes, including thermoregulation and delivery/removal of nutrients and waste products [5].

The time course of post-exercise cardiac autonomic recovery reflects restoration of cardiovascular homeostasis, which is an important component of overall recovery. After exercise, there is a reactivation of parasympathetic modulation [6], which can be measured noninvasively by HR variability (HRV) indices [7]. Importantly, these changes appear to track the time course of homeostasis restoration[8].

The importance of rapid post-exercise cardiac autonomic recovery is reflected by the associations between high post-exercise parasympathetic reactivation with cardioprotection [9], training status [1], [10], and training responsiveness [11]. Several clinical studies have documented a negative relationship between low parasympathetic activity and the progression of cardiovascular disease [12]–[14]. For example, a decrease in parasympathetic and/or an increase in sympathetic HRV indexes have been observed in patients with cardiovascular disease [14]. Thus, sympathetic hyperactivity [15] or reduced vagal tone [16] following exercise may confer a poor cardioprotective background increasing the risk of ventricular fibrillation and sudden cardiac death. Additionally, HRV measurements have more recently been shown to be predictive of changes in aerobic endurance capacity [17], [18]. Indeed, the prescription of exercise training intensities to allow individuals to fully recover their resting HRV indices led to better performance improvement than non-HRV tailored training [11], [18]. Moreover, resting and post-exercise HRV measurements have been shown to be predictive of aerobic performance [17] and improved performance after a training period for different sports [19], [20]. Therefore, strategies to accelerate post-exercise parasympathetic reactivation have received great interest in recent years, and the use of water immersion (WI) is one of the most studied strategies.

WI has been repeatedly shown to be a simple and efficient method for increasing post-exercise parasympathetic activity, as inferred from HRV measurements [10], [21]–[24]. This recovery strategy induces a hydrostatic pressure that shifts peripheral blood into the thoracic vasculature [25], thereby increasing central blood volume and venous pressure, stroke volume, and cardiac output [26]. The increase in central venous pressure stimulates high arterial pressure and low cardiopulmonary pressure baroreflexes [27], [28], which may augment parasympathetic activity [28] and, consequently, increase vagal-related HRV indices [29].

The water temperature may also affect post-exercise parasympathetic reactivation. It is suggested that the water temperature changes peripheral cutaneous vasomotor tone, and consequently, baroreceptor loading, resulting in changes in parasympathetic activity [25]. For instance, Buchheit et al. [22] showed that five min of cold WI (CWI, water temperature ∼ 14°C) after supramaximal exercise was associated with faster parasympathetic reactivation compared with the control condition (no immersion, CTRL). Al Haddad et al. [30] reported that CWI (14–15°C for 5 min) or thermoneutral WI (TWI, 33–34°C for 5 min) were both effective in increasing parasympathetic reactivation after submaximal exercise, but CWI was found to be more effective. Moreover, Stanley et al. [23] demonstrated that CWI (∼ 14°C for 5 min) or contrast WI for 10 min (three sets of 3 min, 14°C for 1 min + 36°C for 2 min followed by another 1 min at 14°C) were equally effective for increasing parasympathetic reactivation following a high-intensity interval exercise.

To the best of our knowledge, no study has investigated the effect of hot WI (HWI, water temperature > 36°C, HWI) [25], [31] on post-exercise parasympathetic reactivation. It is proposed that at rest, HWI increases cutaneous and subcutaneous skin temperatures due to vasodilation [32], [33]. The heart rate will also increase in response to HWI [32], [34] and may reduce stroke volume because of a lack of cardiac filling time, but overall cardiac output increases in comparison with thermoneutral conditions [34]. However, it is difficult to predict parasympathetic reactivation since WI *per se* has an effect on central blood volume expansion and, hence, on autonomic nervous system activity [25], [26]. Therefore, it is not known the effects of HWI on post-exercise parasympathetic reactivation.

Although WI (cold or thermoneutral) increases post-exercise parasympathetic reactivation [22], [30], few studies have evaluated parasympathetic reactivation after WI and the results are conflicting [21], [23]. Stanley et al. [23], for example, found that CWI increased parasympathetic modulation for as long as 3 hours after the exercise session, while Bastos et al. [21] did not observe significant differences in post-exercise parasympathetic reactivation using CWI or passive recovery following approximately 1.5 hours of supine resting. Long-lasting (> 4 hours) increased parasympathetic modulation might be important for competitions and training sessions that occur twice a day (morning and afternoon training sessions) [35]. Therefore, the effect of post-exercise WI using different temperatures on long-lasting parasympathetic reactivation needs further investigation.

Finally, it is also interesting to note that the previous studies applied WI interventions that lasted from 5 to 10 min [21]–[23], [30] and there is evidence that compared to a 6 min protocol, contrast WI for 12 min has greater effect on performance recovery, although the effects on post-exercise parasympathetic reactivation were not evaluated [36]. Thus, it would be interesting to investigate the effect of a longer post-exercise WI period (> 10 min) on parasympathetic reactivation [37].

Therefore, the aim of the present study was to investigate the effect of WI using different temperatures on post-exercise parasympathetic reactivation. We used three WI temperatures (cold – 15°C, temperate – 28°C and hot – 38°C) for 15 min and evaluated the parasympathetic reactivation during and after WI (∼4 hours after the exercise session). We hypothesized that WI *per se* would increase post-exercise parasympathetic reactivation and that, the cooler the water, the greater would be the parasympathetic reactivation. Additionally, we hypothesized that the effects of CWI on HRV indices would last longer than the other strategies, providing evidence for the adoption of this type of post-exercise recovery strategy in sports settings.

## Material and Methods

### Participants

Sample size was calculated based under the assumption that a 0.5 ± 0.3 ms difference in ln rMSSD [30], [38] during WI was meaningful. Using Statistica 10.0 (StatSoft Inc., Tulsa, OK, USA) we determined that eight subjects would provide a statistical power of 80% at an alpha level of 0.05. Thus, the present study enrolled eight non-smokers, healthy and trained young men (mean ± SD; peak oxygen uptake (VO_2_ peak): 54.2 ± 3.6 mL.kg^−1^.min^−1^; age: 24 ± 6 years; height: 176 ± 3.3 cm; body mass: 68 ± 8.6 kg; body mass index: 21.9 ± 2.6 kg.m^−2^; body fat percentage: 6.8 ± 2.5%). Participants were not professional athletes, but they were regularly engaged in various intermittent activities such as soccer, cycling and running, 2 to 5 times a week, each session lasting over 2 hours. Furthermore, as inclusion criteria, subjects should have a VO_2_peak > 50 ml.kg.^−1^.min^−1^. Prior to data collection, a medical questionnaire was completed by the participants to exclude individuals who were taking medications or having recent musculoskeletal injuries. The physical activity readiness questionnaire (PAR-q) [39] was applied to exclude individuals at risk for performing physical activity.

### Ethics Statement

The study was approved by the local human research ethics committee (Centro Universitário de Belo Horizonte – Minas Gerais - Brazil; protocol number 009/2008) and was performed in accordance with the ethical standards of the National Council of Health (resolution 196/96), according to the current Brazilian laws for research involving human subjects. Volunteers were made aware of all experimental procedures and risks by thoroughly explanation before engaging in the research and they signed an informed consent form before participation.

### VO_2_ peak and 1-RM

Prior to the experimental sessions, volunteers had their peak oxygen consumption (VO_2_ peak) and one maximum repetition (1-RM) of knee extension measured. The VO_2_ peak was measured by indirect calorimetry (K4b2, Cosmed) using a customized ramp protocol, performed on a treadmill (PRO 300 RT, Movement, Brazil), as described elsewhere [40]. Briefly, the ramp protocol consisted initially of three minutes of warm-up at 5 km.h^−1^. Then, the speed and grade were increased every 60 seconds at an individual rate until fatigue occurred despite verbal encouragements. The speed increments were based on the volunteer training history to induce fatigue between 8 and 12 min. The 1-RM test was performed as described by Verdijk et al. [41]. Briefly, the 1-RM was measured in four to five trials by successively increasing the load for each lift. Rest periods were 2–3 min between each trial. Approved lifts had to reach 160° in the knee joint.

### Experimental Procedures

**Figure 1:**
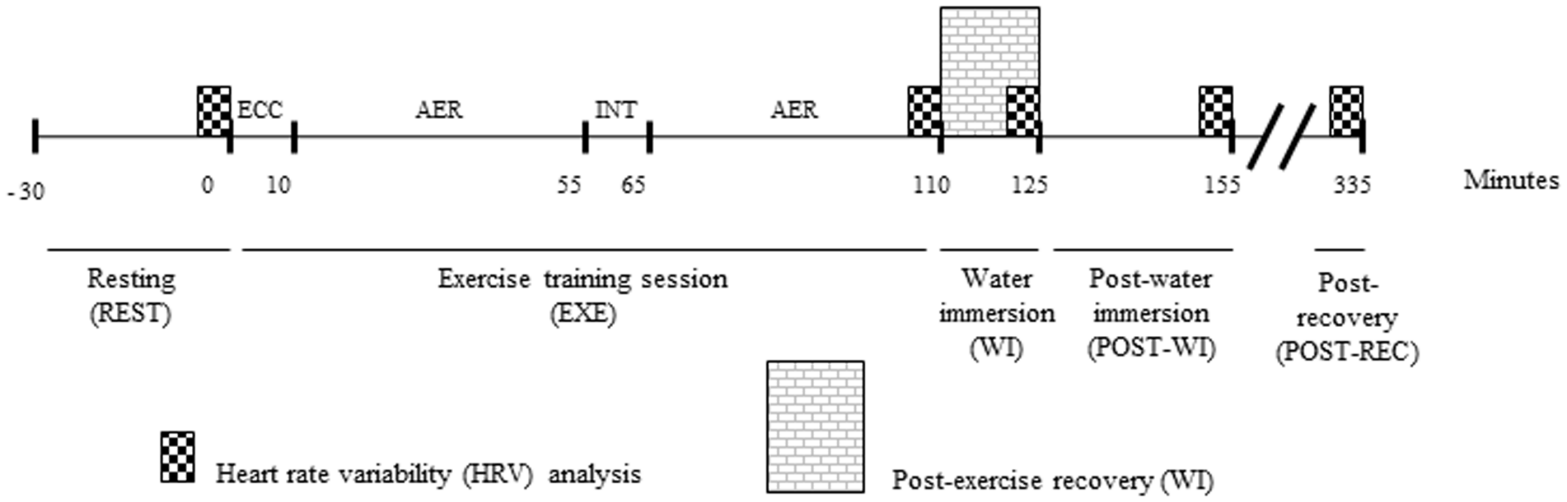
Experimental design employed in the present study. Subjects rested for 30 min (REST) and then performed an exercise training session (EXE) composed of approximately 10 min of a resistance eccentric exercise bout (EC) and a 90 min submaximal aerobic exercise bout (AER) (two 45 min at 70% VO_2_max, interspersed by a 10 min passive rest, INT). The exercise training session was followed by four recovery strategies for 15 min (WI) in a randomized, crossover design: no immersion (CTRL), cold water immersion (CWI), temperate water immersion (TWI) and hot water immersion (HWI). After that, subjects recovered at room temperature for another 210 min (3 h30 min), divided in post-water immersion (POST-WI) and post-recovery (POST-REC). Heart rate variability indices were measured in the last 5 min of REST, WI, POST-WI and POST-REC.

After resting for 30 min in the supine position (REST), subjects completed an exercise session involving resistance exercise and submaximal running (EXE) followed by one of the four recovery strategies for 15 min (WI): no immersion (CTRL), CWI, TWI or HWI, in a randomized fashion. Thereafter, the subjects recovered at room temperature for 30 min POST-WI (30 min after the WI) followed by another 195 min period (POST-REC, total of 240 minutes after the exercise session). HRV indices were measured during the last 5 min of REST, WI, POST-WI and POST-REC periods (Figure 1).

### Exercise session

Subjects were previously instructed to refrain from exercise, alcohol and caffeine consumption during the previous 24 hours. All volunteers recorded their food intake on the night prior to and the day of the first experimental session, and were asked to repeat their ingestion for the following experimental sessions. The successful replication of the food intake was checked upon the arrival of the subject at the laboratory on each experimental day. They were also asked to drink 500 mL of water two hours before arriving at the laboratory.

Each volunteer was required to complete four experimental sessions separated by at least five days (7 ± 2 days) in a randomized, crossover design. Subjects were instructed to arrive at the laboratory in the morning (∼8:00 am). Upon arrival, a urine sample was collected and the volunteers were considered hydrated in all situations (urine specific gravity lower than 1.020 measured by hand-held clinical refractometer, model RHC 200ATC [42]). Then, volunteers inserted a rectal thermistor probe (YSI 401) 12 cm beyond the anal sphincter and were instrumented with a HR transmitter strap (S810i series TM, Polar, USA). The rectal temperature measurements corresponded to the five-minute period when HRV indices were assessed. All the session were conducted in a controlled temperate environment (19.8 ± 1.8°C and 71 ± 9% relative humidity).

Then, the subjects laid in a supine position for 30 minutes in a quiet room. After baseline measurements, they were required to complete an eccentric exercise (ECC; three sets of 10 repetitions of unilateral knee flexion, at 100% of 1-RM measured during the concentric phase). The concentric phase of the movement (knee extension) was performed by two researchers, and subjects performed only the eccentric phase (knee flexion). This ECC protocol was employed to cause neuromuscular overload. This resistance exercise session lasted approximately 10 min. Immediately after the resistance exercise, subjects completed a prolonged moderate intensity continuous running on a motorized treadmill (AER; 2 × 45 minutes at 70% of VO_2_ peak with 10 min of rest between bouts). Treadmill speed was adjusted to guarantee the pre-defined VO_2_ values (70% of VO_2_ peak). Oxygen consumption was measured in the first experimental session of each subject, and the adjustments made in the treadmill speed of the first session were mimicked for the remaining three sessions. This exercise design was chosen to concomitantly stress the cardiovascular and neuromuscular systems and it was also developed for another study in which we investigated the water immersion recovery strategies on muscle damage and exercise performance. Importantly, this exercise design reduced running speed during a 5 km time trial performed 4 hours after recovering at room temperature and increased plasma markers of muscle damage, aspartate aminotransferase and creatine kinase activities (data not shown).

### Recovery strategies

Immediately after the exercise, the volunteers were randomly assigned to recover passively for 15 min in a plastic tank (65 cm radius and 70 cm height) in either WI at 15, 28 or 38°C (±1°C) or CTRL. During WI volunteers remained seated (20° of knee flexion) and were immersed to the xiphoid process level with the arms out of the water wearing only shorts. Before immersion, the desired water temperature was achieved using a thermal resistance (38°C and 28°C) or by crushed ice (15°C). Water was circulated every 3 min to maintain a homogenous temperature that was checked with a thermometer. During CTRL, volunteers remained seated in the plastic tank without water. Immediately after the 15 minutes of WI recovery, the volunteers were immediately toweled dry and rested in a supine position, covered with a blanket, for an additional 30 min. Then, the subjects received a standard diet according to their caloric expenditure during the experimental session, calculated by using commercial available nutrition software (Avanutri Revolution 4.0, Avanutri, Brazil). Water was also given to the subjects to meet their fluid loss. Subsequently, subjects recovered in the laboratory for another 165 min, during which they were allowed to read, use the computer or remain passively seated. They were not allowed to leave the laboratory or to be physically active. Total recovery time (time between the end of exercise and the last measurement) was therefore 240 minutes (4 hours). We choose this design based on sport events or training routines, which individuals train or compete in the morning and afternoon. During the last 15 min of the recovery period, subjects were seated comfortably on a chair and remained quiet and silent.

### Data measurements and analysis

The Polar Electro RS800 HR monitor was used to continuously record beat-to-beat HR, during the entire experimental session. The use of this equipment in heart rate variability (HRV) analysis has been previously validated [43] and is commonly used [44]. Data collected with the HR monitor was transmitted, via an infrared sensor, to a personal computer, equipped with the Polar Pro Trainer 5 software. Data were then digitally filtered (moderate power filter and a protection zone of at least 6 bpm), and only series with more than 95% of the sinus beats were considered for analysis. Signal processing was then performed using HRV analysis software 2.0 (Biomedical The Signal Analysis Group, Department of Applied Physics, University of Kuopio, Finland). The following HRV indices were calculated considering the last 5 min of each of the experimental intervals depicted in figure 1: mean R-R intervals (mR-R), the square root of the mean of the sum of the squares of differences between adjacent normal R–R intervals (rMSSD), instantaneous beat-to-beat variability (SD1) and continuous beat-to-beat variability (SD2), both derived from the Poincaré Plot analysis [45].

### Statistical analysis

Data are reported as mean ± SEM, unless stated otherwise. We had to exclude the POST-REC values of one subject due to data poor quality. For every analysis, data normality was checked by the Kolmogorov-Smirnov & Liliefors test. When normality using the raw data was not observed, data were transformed by taking its natural logarithm and normality then checked again. This happened for rMSSD and SD1; therefore, these data are shown as ln rMSSD and ln SD1, respectively. This procedure allowed parametric statistical comparisons. A two-way repeated measures analysis of variance (ANOVA two-way) was used to compare HRV indices over time and among the four experimental conditions. When a significant F value was observed in the ANOVA, the significance of the difference between the means obtained was determined by Tukey HSD post hoc test. Alpha was set at 0.05. The statistical package Statistica 10.0 was used for the analyses. Data were also analyzed for practical significance using magnitude-based inferences [46]. We used this qualitative approach because traditional statistical approaches often do not indicate the magnitude of an effect, which is typically more relevant to athletic performance than any statistically significant effect. Effect sizes (Cohen's d) were calculated to analyze the potential trends in HRV indices during each moment compared with CTRL. An ES of <0.2 is classified ‘trivial’, 0.2–0.5 as ‘small’, 0.5–0.8 as ‘moderate’ and >0.8 as ‘large’ effect.

## Results

### Heart rate and rectal temperature responses to the exercise bout and post-exercise recovery period

**Figure 2:**
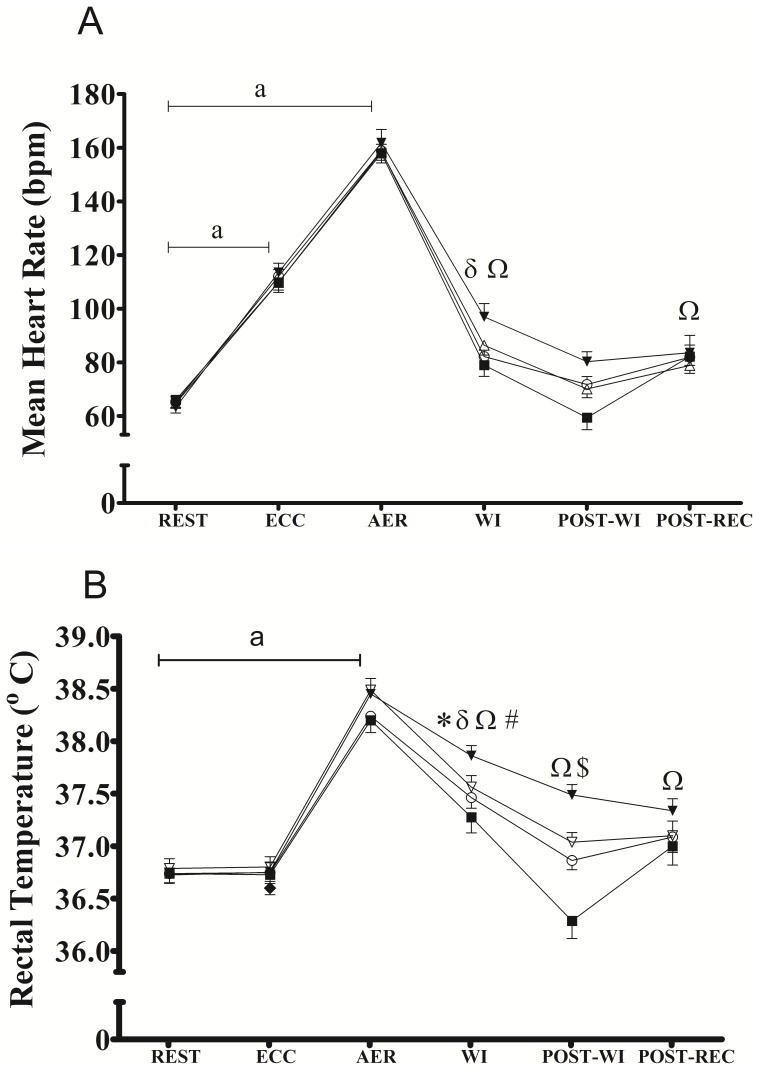
Mean heart rate (panel A) and mean rectal temperature (panel B) at rest (REST), during resistance eccentric exercise (ECC), during aerobic endurance exercise (AER) during post-exercise water immersion (WI), during post-water immersion (POST-WI), and during post-recovery period (POST-REC) for no immersion (CTRL, ο), cold water immersion (CWI, ▪), temperate water immersion (TWI, Δ) and hot water immersion (HWI, ▾). ^a^ p<0.05 vs REST for all recovery strategies; * p<0.05 vs rest in CTRL; ^δ^ p<0.05 vs rest in TWI; ^Ω^ p<0.05 vs rest in HWI; ^#^ p<0.05 CWI vs CTRL; ^$^ p<0.05 HWI vs CTRL. Data are shown as mean and SEM.

Figure 2 shows the heart rate (2A) and rectal temperature (2B) responses. There were no differences (p>0.05) in the mean HR and rectal temperature during rest, and ECC and AER among the conditions, confirming that the exercise bout induced similar cardiovascular and thermoregulatory disturbances in all experimental conditions. During WI, the mean HR was higher for TWI and HWI compared with for REST (figure 2A), but no significant differences were observed among the recovery strategies. During POST-WI, the mean HR was not different for any of the recovery strategies compared with REST (figure 2A). During POST-REC, the mean HR was higher only for HWI compared with REST (figure 2A).

During WI, rectal temperature was higher for CTRL, TWI and HWI compared with REST (figure 2B). During POST-WI, the rectal temperature was higher for HWI compared with REST (p<0.001) and CTRL (p = 0.014) (figure 2B). During POST-REC, only HWI showed increased (p = 0.031) rectal temperature values compared with REST and no differences among the recovery strategies were observed (figure 2B).

### HRV indices during rest and exercise

**Figure 3:**
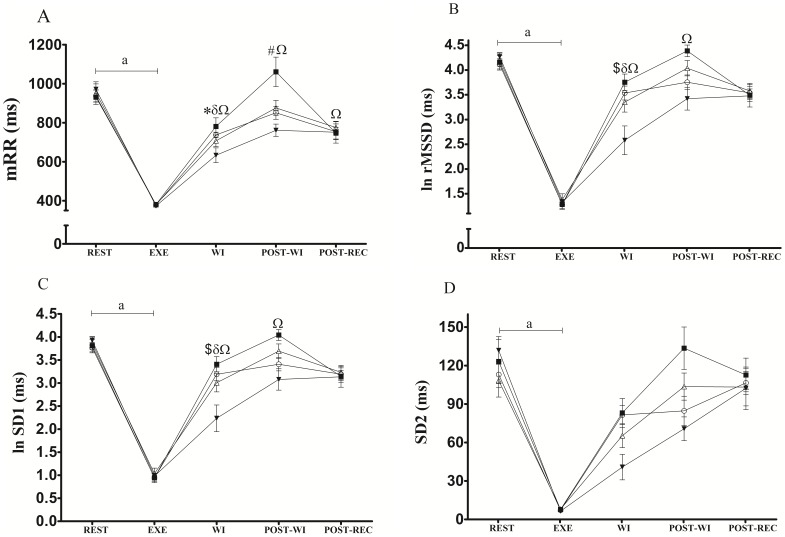
Mean R-R (panel A), ln rMSSD (panel B), ln SD1 (panel C) and SD2 (panel D) at rest (REST), during exercise (EXE), during post-exercise water immersion (WI), during post-water immersion (POST-WI), and during post-recovery period (POST-REC) for no immersion (CTRL, ο), cold water immersion (CWI, ▪), temperate water immersion (TWI, Δ) and hot water immersion (HWI, ▾). ^a^ p<0.05 vs REST for all recovery strategies; * p<0.05 vs rest in CTRL; ^δ^ p<0.05 vs rest in TWI; ^Ω^ p<0.05 vs rest in HWI; ^#^ p<0.05 CWI vs CTRL; ^$^ p<0.05 HWI vs CTRL. Data are shown as mean and SEM.

There were no significant differences in the HRV indices measured at REST or during EXE in all four experimental conditions (figure 3A-D), confirming similar basal conditions before the recovery strategies and similar reductions in HRV indices during the exercise bout.

### HRV indices during WI period

During WI, mRR (figure 3A) was reduced for CTRL, TWI and HWI compared with REST, while for CWI, mRR did not differ from REST. For both ln rMSSD (figure 3B) and ln SD1 (figure 3C), TWI and HWI values were lower relative to REST, and the values for HWI were lower relative to CTRL. For SD2 (figure 3D), we did not observe an interaction between recovery strategies and moment (p = 0.085). However, there was a main effect for moment (p = 0.00001) on WI, resulting in lower values relative to REST.

Moreover, table 1 shows the magnitude-based inferences calculated for WI recovery strategies versus CTRL. During WI, HWI very likely induces lower values for all HRV indices assessed compared with CTRL, while the effects of CWI and TWI are not overall clear.

**Table 1:** Magnitude-based inferences during water immersion (WI), post-WI and after the recovery period (POST-REC) for ln rMSSD, mRR, SD1 and SD2 in cold WI (CWI), temperate WI (TWI) and hot WI (HWI).

	ln rMSSD	mRR	SD1	SD2
**WI**				
**CWI**	Unclear	Unclear	Unclear	Unclear
**TWI**	Unclear	Unclear	Unclear	Likely
**HWI**	Very Likely	Very Likely	Very Likely	Very Likely
**POST-WI**				
**CWI**	Very Likely	Very Likely	Very Likely	Vey Likely
**TWI**	Likely	Unclear	Likely	Unclear
**HWI**	Unclear	Very Likely	Unclear	Possibly
**POST-REC**				
**CWI**	Unclear	Unclear	Unclear	Unclear
**TWI**	Unclear	Unclear	Unclear	Unclear
**HWI**	Unclear	Unclear	Unclear	Unclear

All inferences were calculated comparing the water immersion strategy with the CTRL condition. mRR: mean R-R intervals; ln rMSSD: normal logarithm of the square root of the mean of the sum of the squares of differences between adjacent normal R–R intervals; SD1: instantaneous beat-to-beat variability; SD2: continuous beat-to-beat variability.

Table 2 shows the effect sizes calculated by comparing water immersion strategies versus CTRL for each of the heart rate variability indices and moment. During WI, the effect size ranged from trivial (SD2) to moderate for CWI (the other 3 indices). For TWI, effect sizes were large for SD2 and small for the other 3 indices assessed. For HWI, all HRV indices assessed showed large effect sizes during WI.

**Table 2:** Effect sizes (*Cohen′s d*) calculated by comparing water immersion strategies versus CTRL for each of the heart rate variability index and moment.

	ln rMSSD	mRR	SD1	SD2
**WI**				
**CWI**	0.54	0.60	0.54	0.08
**TWI**	0.41	0.33	0.41	0.91
**HWI**	1.68	1.23	1.68	1.81
**POST-WI**				
**CWI**	1.83	1.38	1.82	1.24
**TWI**	0.71	0.45	0.71	0.56
**HWI**	0.66	1.11	0.66	0.69
**POST-REC**				
**CWI**	0.15	0.24	0.15	0.21
**TWI**	0.11	0.38	0.11	0.12
**HWI**	0.13	0.91	0.13	0.19

Effect sizes <0.2 ‘trivial’; 0.2–0.5 ‘small’; 0.5–0.8 ‘moderate’ and >0.8 ‘large’.

CTRL is control (recovery at room temperature), CWI is cold water immersion (recovery in water at 15°C), TWI is temperate water immersion (recovery in water at 28°C), HWI is hot water immersion (recovery in water at 38°C). WI is the response during water immersion, POST-WI is the response after water immersion, POST-REC is the response after 4 hours of recovery. mRR: mean R-R intervals; ln rMSSD: normal logarithm of the square root of the mean of the sum of the squares of differences between adjacent normal R–R intervals; SD1: instantaneous beat-to-beat variability; SD2: continuous beat-to-beat variability.

### HRV indices during the POST-WI period

During POST-WI, the mRR (p = 0.005, figure 3A), ln rMSSD (p = 0.0089, figure 3B) and ln SD1 (p = 0.0088, figure 3C) were lower for HWI compared with REST. Furthermore, the mRR values for CWI were higher (p = 0.0052, figure 3D) compared with CTRL.

Table 1 shows that during the POST-WI, the magnitude-based inferences of HWI and TWI were not consistent among the indices assessed, although the effects of CWI are very likely higher than CTRL.

Table 2 shows that during the POST-WI moment, CWI showed large effect sizes for all HRV indices, while for TWI the effect sizes ranged from small (mRR and SD2) and moderate (ln rMSSD and SD1). For the HWI condition the effect sizes were large for mRR and moderate for the other 3 indices.

### HRV indices during the POST-REC period

During POST-REC, the HWI showed lower (p = 0.0042, figure 3A) values for mRR compared with REST, while no differences were observed for the other recovery strategies for any of the indices. However, the comparison among the conditions did not show any significant differences at this time point. These results were also observed analysing the magnitude-based inferences (table 1, all indices were unclear) and effect size (table 2) for which ln rMSSD, SD1 and SD2 showed trivial effect sizes for all the WI temperatures. The only exception was mRR that showed a small effect sizes for CWI and TWI and a large effect size for HWI.

## Discussion

In the present study, we aimed to investigate the effects of different WI temperatures on post-exercise parasympathetic reactivation assessed by HRV indices. This is the first study that evaluated a large spectrum of water temperatures (from 15 to 38°C) for a long term recovery period (up to 4 h after the exercise session). The main observed results were: 1) during post-exercise WI, only CWI was able to reestablish all HRV indices relative to REST, while for CTRL, only mRR did not reach REST values; for TWI and HWI only SD2 was reestablished during WI; for HWI; all indices were very likely lower compared with CTRL; 2) during the 30 min POST-WI period, CWI had reestablished all HRV indices and mRR values were higher relative to CTRL; moreover, all HRV indices assessed were very likely higher for CWI than for CTRL; CTRL and TWI had completely reestablished all the HRV indices, and no indices were reestablished in HWI; and 3) during POST-REC, HWI showed reduced values for mRR, but no differences among the other recovery strategies was noticed. Overall, the results indicate that CWI is an effective recovery strategy for parasympathetic reactivation in short term (up to 45 min), but its effects are not evident 4 hours after the exercise bout. Furthermore, HWI in the short-term delays HRV indices recovery after exercise, but in the long term, no difference among WI temperatures are observed.

### Physiological responses to the exercise bout

This study evaluated the effects of different WI temperatures after a combination of eccentric and running submaximal exercises. This exercise protocol was chosen to cause stress on the cardiovascular and neuromuscular systems, and it also resembles the daily activity of different sports modalities where both strength and endurance training are performed concurrently [47]. The cardiovascular and thermoregulatory responses to the exercise bout employed were similar among the conditions, suggesting that differences in the HR and rectal temperature during and after the WI periods were induced by the different recovery strategies applied.

### Effects of WI on HRV parasympathetic reactivation

Our results indicate that CWI is a suitable strategy for inducing fast post-exercise parasympathetic reactivation and that TWI and HWI should not be employed for that specific purpose. Buchheit et al. [22] were probably the pioneers to show that CWI was a better strategy than CTRL to increase parasympathetic reactivation after a supramaximal exercise bout. Increased resting parasympathetic modulation is associated with running performance in a 10 km time trial and with maximal running speed in a maximal running test [10]. Buchheit et al. [10] reported that subjects that had the greatest changes in resting rMSSD after a training program were the ones who showed the greatest increases in running performance. Additionally, the authors also reported that the relative changes in post-exercise parasympathetic reactivation after training were also positively correlated with changes in running performance. These results suggest the possible relationship between performance and post-exercise parasympathetic reactivation.

It has been suggested that the exercise mode [48] and intensity [7] might affect the post-exercise parasympathetic reactivation. Buchheit et al. [7] showed that, after a 6-min repeated sprints session or after a 12-min high intensity interval training session, HRV indices were lower compared with a moderate intensity exercise session (65% VO_2_peak), suggesting that the anaerobic component of the exercise session plays an important role in delaying post-exercise parasympathetic reactivation. In addition, Heffernan et al. [48] found that a session of resistance exercise (10-repetition maximum test) reduced the parasympathetic reactivation of HRV more than an aerobic exercise session (30 min cycling at 65% VO_2_ peak). In the present study, the exercise protocol involved both resistance eccentric exercise and prolonged submaximal running. Our idea was to incorporate exercise components present in a regular training session that could alter significantly the parasympathetic tonus. To the best of our knowledge, the present study is the first to describe the effects of parasympathetic reactivation after a combination of eccentric resistance and aerobic prolonged submaximal exercise with different WI temperatures.

It is interesting to notice that during the immersion period, both TWI and HWI parasympathetic reactivation were decreased compared with REST. This response is contrary to our hypothesis that the immersion *per se* would increase HRV indices, and also contradicts some results found in the literature [30]. At rest, HWI increases body temperature and cardiac output [25]. We are unaware of previous studies that investigated the effects of HWI on post-exercise parasympathetic reactivation. Because WI itself can increase parasympathetic reactivation [30], the effects of HWI on post-exercise parasympathetic reactivation were difficult to predict, but we speculated that the effect of hydrostatic pressure would accelerate parasympathetic reactivation compared with CTRL, even though the temperature effect would lead to delayed parasympathetic reactivation compared to the other immersion strategies. Unexpectedly, TWI also showed delayed parasympathetic reactivation in 3 out of 4 HRV indices assessed in the present study. Using a water bath temperature of 33–34°C, Al Haddad et al. [30] showed that post-exercise parasympathetic reactivation was accelerated compared with CTRL. An explanation for the discrepancies between ours and this previous study might rely on the differences in the exercise protocol employed, as discussed above, and on the effect of WI on rectal temperature and HR. Our results showed that during the immersion period, rectal temperature and HR were higher in TWI and HWI compared with REST. In this regard, we can speculate that during these conditions, cardiac output was increased to meet the needs of the thermoregulatory system for cutaneous vasodilation. This response reduced blood pressure, and consequently caused baroreceptor unloading, increasing the sympathetic tonus and delaying parasympathetic reactivation. Also, the hydrostatic effects related to WI might have been insufficient to increase parasympathetic reactivation. In the study of Al Haddad et al. [30] subjects were immersed to the mid-sternal level in comparison to the xiphoid process immersion of the current study, although we do not think this small difference in immersion depth would account for the differences in the results between the studies. Although Al Haddad et al. [30] did not report the core temperature in their study, the mRR reported was higher for the 33–34°C WI condition compared with their CTRL. In the present study, during WI we did not find a significant difference in mRR between TWI and CTRL. However, a closer inspection of Al Haddad et al. [30] results reveals that ln rMSSD and SD1/SD2 ratio were not different between 33–34°C WI and CTRL, suggesting that the effect of 33–34°C WI strategy on post-exercise parasympathetic reactivation is somewhat limited and not consistent across the HRV indices assessed. This is similar to what we observed in the present study (a similar effect of TWI and CTRL conditions on HRV indices), and, thus, our results agree with their previous observations. Therefore, even though some differences in the exercise protocol used and the mRR values observed may differ between the present and this previous study [30], overall there is agreement between the results. In summary, the results of the present study suggest that post-exercise TWI and HWI are counterproductive for reestablishing post-exercise parasympathetic activity.

### Effects of post-exercise recovery strategies on short-term (POST-WI) and long-term (POST-REC) recovery on parasympathetic reactivation

Although a rapid increase in post-exercise parasympathetic reactivation is important, and WI has been shown to be a simple and time-efficient strategy to do so [22], [30], it would also be important to maintain parasympathetic activation for as long as possible, because increased HRV indices have been reported to predict exercise performance of athletes and non-athletes [10], [24]. Additionally, after a training program, subjects with greater percentage of parasympathetic index increase showed greater improvements in aerobic fitness [10].

In this context, very few studies have assessed the effects of WI on parasympathetic reactivation after the immersion period [21], [23], [24]. Similar to our results, Parouty et al. [24] observed higher parasympathetic activity after CWI than CTRL after a maximal 100 m swimming test. However, parasympathetic reactivation was measured immediately after the CWI period for only 5 min. In the present study, we expanded the period evaluated assessing those indices for 30 min after the 15 min WI period. Bastos et al. [21] studied the effects of CWI after a maximal exercise bout for 83 min on rMSSD and detected a statistically non-significant moderate effect size favoring CWI at 8 and 23 min compared with CTRL. In contrast, Stanley et al. [23] studied the effects of CWI for 20 min after a high-intensity interval training session on ln rMSSD and showed that CWI induced higher parasympathetic activity throughout the 3 hours post-WI relative to CTRL. Our results agree with Stanley et al. [23] findings, as we showed that in the short-term recovery period (POST-WI), CWI was more effective than CTRL for inducing post-exercise parasympathetic reactivation. Also, after approximately 3 h10 min after the exercise session, Stanley et al. [23] showed that ln rMSSD had returned to pre-exercise values for the CWI, while we showed the same results after 4 h of recovery. However, differently from Stanley et al. [23], after the POST-REC we observed no difference in HRV indices among the recovery strategies, while they observed that ln rMSSD for CWI was likely higher comparing with CTRL. They also observed that ln rMSSD values were likely lower for CTRL compared with pre-exercise values, while we observed that CTRL had reached REST values after the POST-REC. Bastos et al. [21] showed that, after 83 min of the exercise bout, CTRL or CWI did not present any difference in HRV indices compared with CTRL. Our results expand Bastos et al. [21] findings, showing that in the long-term (15 min of WI followed by 3 h45 min of passive recovery), no difference among the recovery strategies is noticed. Unfortunately, we did not find any study that assessed post-WI effects on HRV indices using different water temperatures. To date, however, Al Haddad et al. [35] suggested that using CWI following every training session over a typical training week in swimmers can help maintaining resting HRV higher when compared to training without the use of any recovery method. In contrast, however, Yamane et al. [49] showed that chronic CWI attenuated resistance and endurance training-induced adaptations in previously sedentary subjects. In face of these discrepant results, whether the chronic use of this recovery strategy is related to increase in performance or training intensity, requires further investigation.

Interestingly, HRV indices seemed to follow the changes in rectal temperature observed in the present study. As a general observation, whenever rectal temperature was elevated compared with REST, HRV indices were reduced and vice-versa. This fact is particularly true for HWI, but a trend can be identified in all recovery strategies. Moreover, we did not observe the hydrostatic effect on HRV indices, as the HRV indices were not increased in TWI compared with CTRL. This observation might suggest that the thermoregulatory demands imposed on the body were related to the parasympathetic activation of the sinus node, and that they overcame the hydrostatic pressure stimulus for modulating HRV. However, we cannot exclude the hydrostatic effect from the temperature effect because they might have interacted in order to modulate the HRV. Studies aiming to assess the hydrostatic and temperature effects separately on post-exercise recovery strategies might shed a light on this question. Therefore, using the different WI temperatures employed in the present study, we were able to demonstrate the beneficial effect of reducing body temperature [50], [51] and the detrimental effect of increasing body temperature on post-WI vagal-related HRV indices. However, we cannot exclude the influence of the interaction between reduced or increased rectal temperature and the hydrostatic pressure in modulating the responses observed in HRV indices.

### Limitations

The present study has some limitations worth mentioning. During REST and POST-WI, the subjects were in the supine position, while during WI and POST-REC, they were seated. Body position is known to influence on HRV indices [52]. However, even with these differences in body position, no significant differences were observed for CTRL, TWI and CWI relative to REST and POST-REC. The only condition where HRV indices were different from REST and POST-REC was for HWI, a condition in which rectal temperature was also significantly elevated relative to REST in POST-REC compared with REST. Therefore, the difference in HRV indices observed for HWI might be related to changes in body temperature, and not to body position.

### Practical implications

The present results suggest that, for increasing short-term post-exercise vagal-related HRV indices, subjects should be immersed in cold water. The results also suggest that HWI should be discouraged if the purpose of the strategy is to increase parasympathetic reactivation in the short post-exercise recovery period. However, no difference among the four different post-exercise WI strategies was noticed 4 hours after the exercise bout.

## Conclusions

We conclude that after a combination of resistance and prolonged endurance exercise, resting at room temperature or being immersed at water temperature of 28°C for 15 min followed by 30 min of rest completely reactivates parasympathetic modulation to the heart, while cold water immersion (15°C) very likely induces even greater parasympathetic reactivation compared with CTRL. Conversely, hot water immersion blunts post-exercise parasympathetic reactivation. Additionally, 4 hours after the exercise bout, no difference among the recovery strategies in HRV was noticed suggesting that these strategies have short-lasting effects on post-exercise parasympathetic reactivation. Whether these differences in HRV indices in a short term are translated into improved performance or greater recovery perception requires further investigation.
